# Experimental study of the supercritical CO_2_ diffusion coefficient in porous media under reservoir conditions

**DOI:** 10.1098/rsos.181902

**Published:** 2019-06-05

**Authors:** Junchen Lv, Yuan Chi, Changzhong Zhao, Yi Zhang, Hailin Mu

**Affiliations:** Key Laboratory of Ocean Energy Utilization and Energy Conservation Ministry of Education, School of Energy and Power Engineering, Dalian University of Technology, Dalian 116024, People's Republic of China

**Keywords:** diffusion coefficient, supercritical CO_2_, porous media, reservoir condition, empirical correlation

## Abstract

Reliable measurement of the CO_2_ diffusion coefficient in consolidated oil-saturated porous media is critical for the design and performance of CO_2_-enhanced oil recovery (EOR) and carbon capture and storage (CCS) projects. A thorough experimental investigation of the supercritical CO_2_ diffusion in *n*-decane-saturated Berea cores with permeabilities of 50 and 100 mD was conducted in this study at elevated pressure (10–25 MPa) and temperature (333.15–373.15 K), which simulated actual reservoir conditions. The supercritical CO_2_ diffusion coefficients in the Berea cores were calculated by a model appropriate for diffusion in porous media based on Fick's Law. The results show that the supercritical CO_2_ diffusion coefficient increases as the pressure, temperature and permeability increase. The supercritical CO_2_ diffusion coefficient first increases slowly at 10 MPa and then grows significantly with increasing pressure. The impact of the pressure decreases at elevated temperature. The effect of permeability remains steady despite the temperature change during the experiments. The effect of gas state and porous media on the supercritical CO_2_ diffusion coefficient was further discussed by comparing the results of this study with previous study. Based on the experimental results, an empirical correlation for supercritical CO_2_ diffusion coefficient in *n*-decane-saturated porous media was developed. The experimental results contribute to the study of supercritical CO_2_ diffusion in compact porous media.

## 1. Introduction

Global warming caused by the excessive emission of greenhouse gases (mainly CO_2_) has recently attracted much attention. Many techniques have been proposed to mitigate CO_2_ emissions [1–3]. Carbon capture and storage (CCS) is one of the most promising options to reduce the atmospheric CO_2_ concentration [4–6]. Injection of CO_2_ into oil reservoirs known as CO_2_-enhanced oil recovery (EOR) can not only help to store the CO_2_ but also improve the recovery of crude oil [7,8]. Hence, this process is considered to be the most cost-effective CCS technique and has been widely used in the development of oilfields [9–11].

During the CO_2_-EOR process, the diffusion of CO_2_ into crude oil results in volumetric expansion and viscosity reduction of oil in the reservoir [12–14]. Therefore, the determination of CO_2_ diffusion coefficient in the porous media saturated with oil has extremely important guidance on CO_2_-EOR risk assessment, engineering design and economic evaluation [15–17].

Many studies have been conducted on the diffusion coefficient measurement using the pressure volume temperature (PVT) method since the 1930s [18–22]. The PVT method is usually coupled with pressure decay method to determine the diffusion coefficient. A PVT cell with constant volume is used as diffusion cell. The pressure of the system is measured and recorded in real time. A pressure–time profile is then obtained and applied with different mathematical models to predict the diffusion coefficient. Renner proposed measuring the diffusion coefficient of CO_2_ and ethane in consolidated porous media using a novel *in situ* method [15]. The experimental diffusion coefficients of CO_2_ and ethane in decane were determined for conditions of 5.86 and 4.14 MPa at 311.15 K, respectively. Riazi used a constant volume diffusion cell to measure the diffusion coefficients of the methane-*n*-pentane system at a constant temperature of 310.95 K [18]. The pressure–time profile was obtained as the CH_4_ diffused into the liquid phase. The pressure decay method was applied to measure the diffusion coefficient. Unatrakarn *et al*. used a similar technique to measure the CH_4_ and CO_2_ diffusion coefficients in porous media and bulk oil phase at pressures up to 3.2 MPa [23]. Li and Dong proposed both experimental and mathematical methods to determine the CO_2_ diffusion coefficient in oil-saturated Berea cores [24]. The CO_2_ diffusion coefficients were measured under a pressure range from 2.3 to 6.5 MPa. Li *et al*. used a similar method to measure the diffusion coefficient of CO_2_ in cores saturated with oil obtained from the Shengli Oilfield in their experiments [25]. The temperature and permeability conditions were approximately 403.15 K and 9 mD, respectively. The experimental pressures in previous studies were usually less than 6 MPa, which is not consistent with the actual formation conditions.

In the present study, a comprehensive experimental investigation was conducted to investigate the effects of temperature, pressure, permeability, CO_2_ state and porous media on the supercritical CO_2_ diffusion coefficient in porous media saturated with *n*-decane. Experimentally, the pressure data for supercritical CO_2_ diffusion process in *n*-decane-saturated Berea core of 23 experiments at elevated temperatures and pressures were measured. The ranges of temperature and pressure were from 10 to 25 MPa and 333.15 to 373.15 K, respectively. Theoretically, a simplified mathematical model of Fick's Law for the diffusion process in porous media was employed to determine the diffusion coefficients of all 23 experiments. Moreover, a pressure–temperature–viscosity-based empirical equation for the supercritical CO_2_ diffusion coefficient in hydrocarbon-saturated porous media was developed. The main objective of this work was to contribute to the experimental study of supercritical CO_2_ transport in consolidated porous media under reservoir conditions by providing exhaustive experimental data.

## 2. Experimental section

### 2.1. Materials

Berea cores with different permeabilities of 50 and 100 mD were used to examine the effect of permeability on the diffusion coefficient. The porosities of the 50 mD core and 100 mD core were 16% and 22.5%, respectively. The top and bottom faces of the core were sealed by epoxy resin to ensure that the diffusion process only occurred from the radial direction through the side face. Pure CO_2_ gas (99.999%) was purchased from Dalian Special Gas Co. Ltd, China. Pure *n*-decane (98%) was used to represent the oil phase and was purchased from Macklin Biochemical Co., Ltd, China.

### 2.2. Experimental set-up

**Figure 1.**
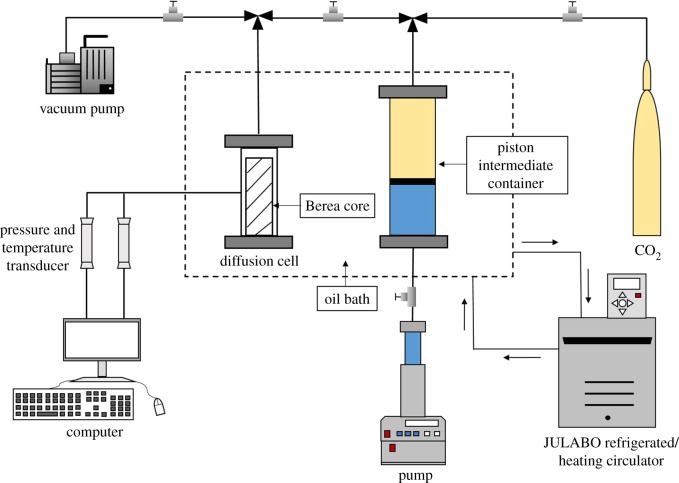
Schematic of the supercritical CO_2_ diffusion experimental set-up.

Figure 1 represents the schematic diagram of the experimental set-up used in this study. An oil bath (CORID CD series, JULABO Inc., Germany) with an accuracy of ±0.03 K was used to control and maintain the temperature of the diffusion cell at the desired value. The pump (D250 L, Jiangsu Haian Oilfield Scientific Instrument Co., Ltd) is applied to syringe highly pressurized supercritical CO_2_ into diffusion cell via intermediate containers and control the pressure of the entire PVT system. The temperatures of the diffusion cell were measured by a temperature transducer with an accuracy of ±0.2 K (JM618I, Jinming Instrument Co., Ltd, China). The pressures were measured using a transducer with an accuracy of ±0.02 MPa (UNIK 5000, GE Druck Ltd, Germany). The change of pressure in the diffusion cell was recorded by a computer in real time.

### 2.3. Experimental process

The measurements of the supercritical CO_2_ diffusion in *n*-decane-saturated Berea cores were performed with the experimental set-up shown in figure 1. The experimental steps were as follows:

(1) The Berea core was fully immersed in a beaker full of *n*-decane and was evacuated for 24 h with a vacuum pump to ensure that the core was fully saturated by *n*-decane.
(2) After the core was positioned in the diffusion cell, the diffusion cell was evacuated for 15 min by a vacuum pump to ensure a vacuum state was reached inside.
(3) The diffusion cell was then heated in the oil bath until the cell temperature reached the desired value.
(4) The CO_2_ in the intermediate container was pressurized to a value 50% higher than the required value for experiments to ensure that the pressure inside the diffusion cell could reach the desired value quickly.
(5) The supercritical CO_2_ was charged into the diffusion cell until the desired pressure was reached. The pressures of the diffusion process were measured by the pressure transducer and recorded by pre-installed software in real time.
(6) After the pressure of the diffusion cell reached steady state, the diffusion process was stopped. The remaining CO_2_ was discharged from the diffusion cell. Then, both the core and diffusion cell were washed and dried to prepare for the next experiment.

## 3. Mathematical formulation

### 3.1. Assumptions

The following are the assumptions proposed for the mathematical model.

(1) In the experiments, the Berea cores were isotropic and *n*-decane was distributed uniformly in it.
(2) The effect of *n*-decane swelling was neglected in the experiments.
(3) During the diffusion process, the supercritical CO_2_ diffusion coefficients were constant in the Berea cores.

### 3.2. Mathematical model

Based on the treatments to the cores mentioned previously, the diffusion process in this study became a natural convection mass transfer process induced by the diffusion of CO_2_ into a liquid-saturated vertical porous column. The effective CO_2_ diffusion coefficient under non-swelling conditions can be obtained from Fick's first Law and the continuity equation, as follows [25]:

3.1 $$\begin{array}{rl} \frac{\partial C}{\partial t}= & D{{\;}^{\prime }}_{\mathrm{eff}}\left(\frac{\partial^{2}C}{\partial r^{2}}+\frac{1}{r}\frac{\partial C}{\partial t}\right),0<r<r_{0},t\geq 0,\\ C{|}_{t=0}= & 0,0<r<r_{0}\\ \mathrm{and}\;C{|}_{r=r_{0}}= & C_{0},t\geq 0, \end{array}\}$$

where *C* denotes CO_2_ concentration in the porous media, mol m^−3^; *r* denotes CO_2_ diffusion radius, 0 < *r* < *r_0_*, m; *r*_0_ denotes the core radius, m; *t* denotes CO_2_ diffusion time, *t* ≥ 0, s; and $D{{\;}^{\prime }}_{\mathrm{eff}}$ denotes CO_2_ diffusion coefficient, m^2^ s^–1^.

A simplified expression of the CO_2_ diffusion coefficient under non-swelling conditions is shown in equation (3.2), which is obtained from other studies [26,27].

3.2 $$D{{\;}^{\prime }}_{\mathrm{eff}}=\frac{\pi }{16}{\left(\frac{r_{0}kV}{N_{\infty }ZRT}\right)}^{2},$$

where *k* denotes the slope of straight line in the coordinate of pressure drop versus square root of time, as shown in figure 3*b*; *V* denotes the annular volume between the core sample denotes gas constant, 8.314 J mol^−1^ K^−1^; *N_∞_* denotes CO_2_ mass diffused into the core when the diffusion time tends to infinity, mol.

## 4. Results and discussion

### 4.1. Validation of the experimental procedure

**Figure 2.**
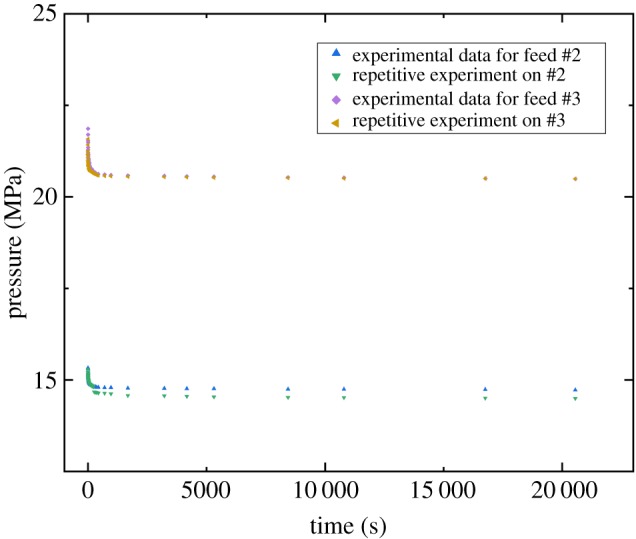
Repetitive experimental data.

Figure 2 presents the results of the reproducibility test for the supercritical CO_2_ diffusion process under pressures from 10 to 20 MPa and temperature at 333.15 K. Figure 2 shows that the original experiments were in good agreement with repetitive experiments. Similar tests were also used in other study to validate the reliability of the experimental apparatus [28].

**Figure 3.**
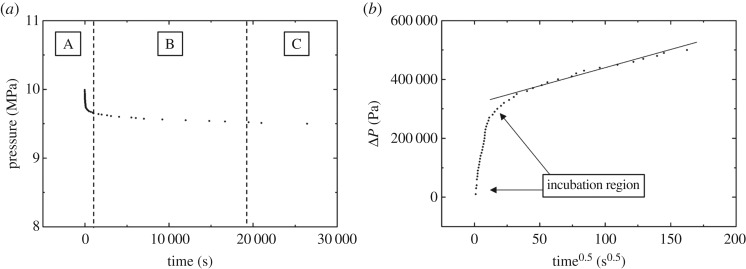
(*a,b*) Experimental data for Experiment No. 1

**Figure 4.**
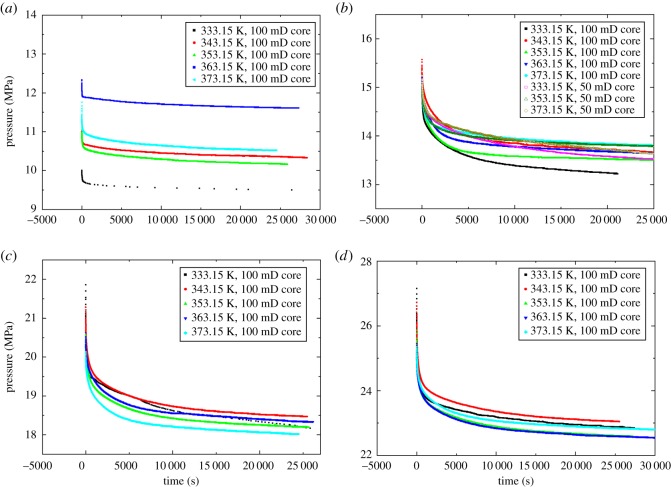
Pressure data from diffusion experiments with different initial pressures: (*a*) 10, (*b*) 15, (*c*) 20 and (*d*) 25 MPa.

The pressure data recorded during the supercritical CO_2_ diffusion process for Experiment No. 1 are shown in figure 3. Figure 3*a* shows that the pressure–time (P–T) profile can be separated into three regions A, B and C corresponding to the rapid pressure decay region, the transition region and the steady pressure region, respectively. The steady state is indicated by the straight line following the inflection point in figure 3*b* which is a plot of the pressure drop as a function of the square root of time. Figure 4 presents the experimental pressure data for Experiments 1–23. Figure 4*b* includes the three diffusion experiments using the 50 mD Berea cores. The remaining experiments used cores with a permeability of 100 mD.

Single factor design was employed to study the effect of temperature, pressure and permeability on the supercritical CO_2_ diffusion coefficient in *n*-decane. In each experiment, only one experimental condition was changed while the other two conditions were kept constant. All the experimental results are listed in table 1.

**Table 1.** Summary of CO_2_ diffusion coefficients and corresponding experimental conditions.

experiments #	pressure (MPa)	temperature (K)	permeability (mD)	decane viscosity (cp)	diffusion coefficients (10^−10^ m^2^ s^−1^)
1	10.00	333.44	100	609.51	0.64
2	15.29	333.24	100	645.67	2.01
3	21.86	333.29	100	691.28	6.30
4	27.15	333.61	100	728.55	8.25
5	11.00	343.53	100	553.31	0.66
6	15.58	343.40	100	581.61	2.23
7	21.37	343.29	100	617.70	6.52
8	26.72	343.35	100	651.42	8.95
9	11.05	353.24	100	500.36	0.94
10	15.04	353.20	100	522.81	3.39
11	20.58	353.22	100	554.05	7.19
12	25.87	353.47	100	584.17	9.78
13	12.33	362.89	100	461.48	1.20
14	15.21	363.15	100	476.3	5.72
15	21.37	363.06	100	508.13	9.54
16	25.45	363.23	100	529.35	11.68
17	11.89	373.12	100	419.72	1.97
18	14.99	372.86	100	434.43	7.97
19	20.14	373.10	100	458.98	11.13
20	25.35	373.45	100	483.84	13.24
21	15.43	333.30	50	646.65	0.88
22	15.03	353.31	50	522.69	1.59
23	15.11	373.04	50	435.02	3.16

### 4.2. Empirical correlation for diffusion coefficient

**Figure 5.**
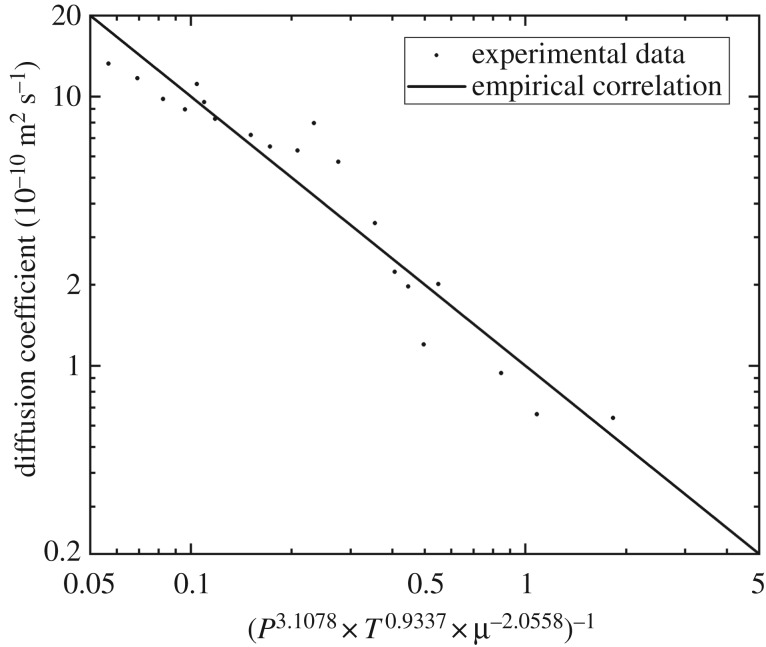
Comparison of experimental results with empirical correlation prediction.

According to the experimental results above, a pressure–temperature–viscosity-based empirical correlation was developed for the supercritical CO_2_ diffusion coefficient in porous media saturated with *n*-decane. The correlation for Berea cores with a permeability of 100 mD is shown in equation (4.1). The R-square of this correlation is 0.9253. Figure 5 shows the comparison between the experimental results and the empirical correlation predictions.

4.1 $$D=10^{-10}\times P^{3.1078}\times T^{0.9337}\times \mu^{-2.0558},$$

where *D* denotes the diffusion coefficient; *P* denotes the pressure, MPa; *T* presents the temperature, K; *μ* is hydrocarbon viscosity, cp.

### 4.3. The effect of pressure

**Figure 6.**
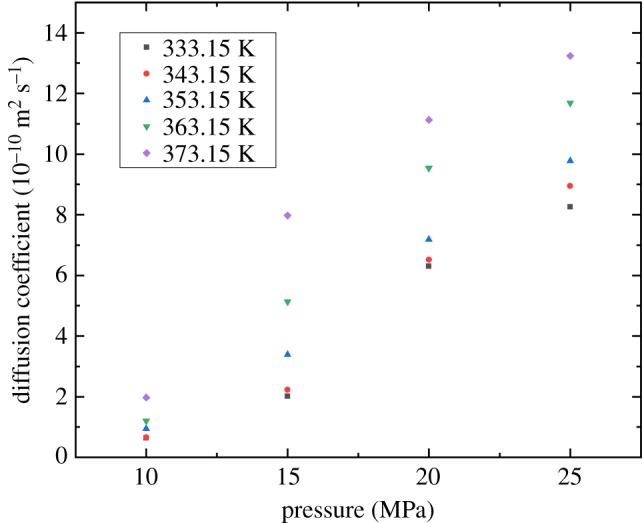
Effect of pressure on the CO_2_ diffusion coefficient.

Figure 6 shows that the diffusion coefficient of the supercritical CO_2_ increases with pressure. The diffusion coefficient at 25 MPa is 12.99 times larger than the one at 10 MPa in 333.15 K experiments. For the remaining four experimental temperatures from 343.15 K to 373.15 K, the diffusion coefficients increase by 13.47, 10.38, 9.72 and 6.70 times, respectively. This shows that the diffusion process will happen faster when the pressure is larger. The concentration of supercritical CO_2_ in the diffusion cell is larger under higher pressure conditions. Thus, the diffusion coefficients increase with the supercritical CO_2_ concentration due to a reduced viscosity of the supercritical CO_2_–decane mixture. Similar experimental observations could be found in other studies [29,30].

### 4.4. The effect of temperature

**Figure 7.**
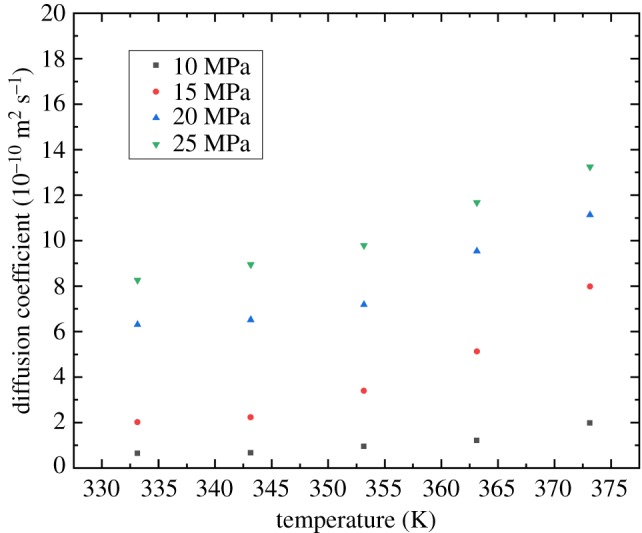
Effect of temperature on the CO_2_ diffusion coefficient.

Figure 7 shows the temperature effect on the supercritical CO_2_ diffusion coefficient. The relationship between the diffusion coefficient and temperature is a parabola that is concave up. The thermal molecular motion is the dominant factor that affected the diffusion process. With increasing temperature, the molecular motion becomes more active so that the CO_2_ diffusion process is enhanced.

### 4.5. The effect of permeability

**Figure 8.**
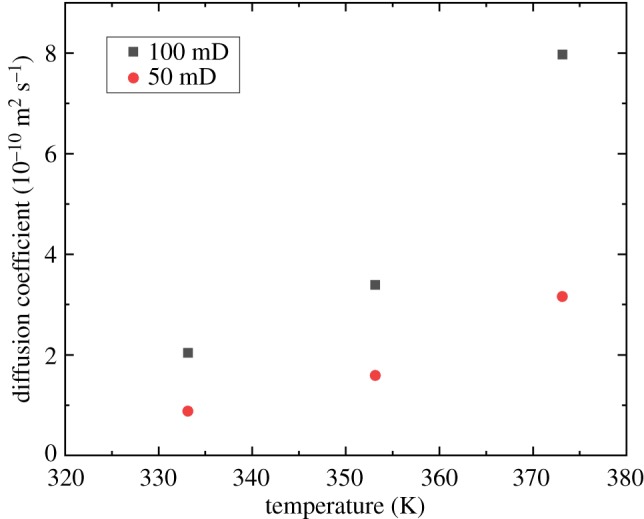
Effect of permeability on the CO_2_ diffusion coefficient.

Figure 8 shows that the diffusion coefficient increases with permeability. The measurements were performed under 15 MPa for all six experiments shown in figure 8. The diffusion coefficient increases by 2.28, 2.13 and 2.52 times at 333.15, 353.15 and 373.15 K, respectively. The permeability of the core has a reciprocal relationship with its tortuosity. As a reflection on the diffusion coefficient, the increase in the permeability indicates a decrease in the tortuosity, which allows the supercritical CO_2_ to flow more smoothly in the cores. As a result, the diffusion coefficient increases with the permeability.

### 4.6. CO_2_ state and porous media effect

The experimental results of the CO_2_ diffusion coefficient in different mixture systems and experimental conditions are listed in table 2. It is noted that the CO_2_ diffusion coefficients from the previous study are generally larger than the results in this study. The differences in results were caused by the state of CO_2_ in the different experiments. The CO_2_ was in the vapour phase in both Grogan *et al.*'s and Bagalkot & Hamouda's studies [31,32]. In our study, CO_2_ was in supercritical state with a larger viscosity and density. The diffusion process was inevitably restrained, which led to a decrease in the diffusion coefficient.

**Table 2.** CO_2_ diffusion coefficient under different conditions.

mixture	experiment condition	diffusion coefficient (10^−10^ m^2^ s^−1^)	reference
CO_2_ + *n*-decane (porous media)	311 K, 1.44–5.83 MPa	104–126	[15]
CO_2_ + bulk *n*-decane	298.15–318.15 K, 2.5–6 MPa	12.1–22.6	[31]
CO_2_ + bulk *n*-heptane	298.15–318.15 K, 2.5–6 MPa	12.9–26.9	
CO_2_ + bulk *n*-hexane	298.15–318.15 K, 2.5–6 MPa	17.4–34.3	
CO_2_ + bulk pentane	298.15 K, 1.54–3.51 MPa	37.2–75.9	[32]
CO_2_ + bulk decane	298.15 K, 1.36–5.63 MPa	18.7–57.1	
CO_2_ + bulk hexadecane	298.15 K, 2.26–5.28 MPa	18.0–31.7	
CO_2_ + bulk *n*-decane	298.15–323.15 K, 1–30 MPa	25–48	[33]
CO_2_ + bulk octane	290–311 K, 1.265–3.103 MPa	2.789–8.105	[34]
CO_2_ + bulk *n*-tetradecane	290–311 K, 0.910–4.041 MPa	0.767–3.731	
CO_2_ + *n*-decane (porous media)	333.15–373.15 K, 10–25 MPa	0.64–13.24	this study

In Moultos *et al.*'s study, the CO_2_ diffusion coefficients in bulk decane were obtained under similar experimental conditions [33]. The diffusion coefficient was 40 × 10^−10^ m^2^ s^−1^ under 323.15 K and 30 MPa condition. The CO_2_ diffusion coefficient in porous media saturated with decane obtained in this study was 8.25 × 10^−10^ m^2^ s^−1^ under 333.61 K and 27.15 MPa condition. Under similar temperature and pressure conditions, the diffusion coefficient in bulk decane is 4.84 times larger than that in porous media. The result clearly shows that the CO_2_ diffusion process is impeded significantly by the presence of porous media. The experimental results in this paper have better practical meaning since the pressure and temperature conditions simulated the underground reservoir conditions.

## 5. Conclusion

In this study, a comprehensive experimental investigation on the effects of the pressure, temperature, permeability, CO_2_ state and porous media on the supercritical CO_2_ diffusion coefficients was carried out. Overall, 23 experiments were performed at pressure ranging from 10 to 25 MPa and temperature ranging from 333.15 to 373.15 K, which simulated the real reservoir conditions. Among them, Experiments 1–20 used Berea cores with a permeability of 100 mD and Experiments 21–23 used cores with a permeability of 50 mD to study the effect of the permeability on the diffusion coefficient.

These results demonstrated that under reservoir conditions the supercritical CO_2_ diffusion coefficient in oil-saturated porous media increased with increasing pressure and temperature, respectively. However, the effect of pressure on the diffusion process decreased at elevated temperature condition. Also, the supercritical CO_2_ diffusion coefficient first increases slowly at 10 MPa and then grows significantly with increasing pressure. The increase in the core permeability increased the diffusion coefficient and the growth trend was steady throughout the whole temperature range in the experiments. The supercritical state of the CO_2_ and the presence of porous media significantly impeded the diffusion process, compared with the results from pure gas CO_2_ diffusion in bulk alkanes in previous study. The pressure–temperature–viscosity-based empirical equation developed in this paper successfully predicted the supercritical CO_2_ diffusion coefficients in porous media under reservoir conditions. Moreover, all the experimental data contributed to the theoretical study of supercritical CO_2_ diffusion in porous media at elevated temperatures and pressures.

## Supplementary Material

Reviewer comments

## Data accessibility

The measured experimental data are available from the Dryad Digital Repository: https://doi.org/10.5061/dryad.n0bf69r [35].
